# Translation factor and RNA binding protein mRNA interactomes support broader RNA regulons for posttranscriptional control

**DOI:** 10.1016/j.jbc.2023.105195

**Published:** 2023-08-24

**Authors:** Christopher J. Kershaw, Michael G. Nelson, Lydia M. Castelli, Martin D. Jennings, Jennifer Lui, David Talavera, Chris M. Grant, Graham D. Pavitt, Simon J. Hubbard, Mark P. Ashe

**Affiliations:** 1 Division of Molecular and Cellular Function, School of Biological Sciences, The University of Manchester, Manchester, UK; 2 Division of Cardiovascular Sciences, School of Medical Sciences, The University of Manchester, Manchester, UK

**Keywords:** mRNA, translation initiation, mRNA decay, RNA binding proteins, RIP-sequencing (RIP-seq)

## Abstract

The regulation of translation provides a rapid and direct mechanism to modulate the cellular proteome. In eukaryotes, an established model for the recruitment of ribosomes to mRNA depends upon a set of conserved translation initiation factors. Nevertheless, how cells orchestrate and define the selection of individual mRNAs for translation, as opposed to other potential cytosolic fates, is poorly understood. We have previously found significant variation in the interaction between individual mRNAs and an array of translation initiation factors. Indeed, mRNAs can be separated into different classes based upon these interactions to provide a framework for understanding different modes of translation initiation. Here, we extend this approach to include new mRNA interaction profiles for additional proteins involved in shaping the cytoplasmic fate of mRNAs. This work defines a set of seven mRNA clusters, based on their interaction profiles with 12 factors involved in translation and/or RNA binding. The mRNA clusters share both physical and functional characteristics to provide a rationale for the interaction profiles. Moreover, a comparison with mRNA interaction profiles from a host of RNA binding proteins suggests that there are defined patterns in the interactions of functionally related mRNAs. Therefore, this work defines global cytoplasmic mRNA binding modules that likely coordinate the synthesis of functionally related proteins.

The translation of mRNA sequence into protein presents the cell with a rapid and direct means to modulate cell physiology by altering the cellular proteome without necessarily requiring new transcript synthesis (1). mRNAs are recruited to the translation machinery by virtue of a highly conserved series of RNA and protein interactions (2, 3). However, alternative mRNA fates are possible. For instance, mRNAs can be stored to enter the pool of translationally active mRNAs when required (4, 5). mRNAs can be targeted for degradation or they can be specifically localized (6, 7). Indeed, the localization of mRNAs within the cytoplasm can influence their translation, storage, and degradation (8). Consequently, the fate of an mRNA in the cytoplasm is complex and highly regulated.

While a range of biochemical and genetic strategies have uncovered the basal translation machinery (2, 3), the question as to how an individual mRNA is selected for translation from the complex pool of transcripts is quite poorly understood. It is well-established that modifications at the 5′ and 3′ ends of an mRNA, the 5′ cap and 3′ poly(A) tail structures, play key roles in translation and protect mRNAs from degradation by virtue of interaction with specific translation initiation factors (9). In addition, a range of RNA binding protein (RBPs) can influence the fate of an mRNA by enhancing or repressing the recruitment of mRNAs to the translation machinery (10). Nevertheless, how the resulting network of RNA and protein interactions is coordinated across the thousands of mRNA molecules within the cell to precisely regulate the scale of translation for each mRNA species is unknown.

Fundamentally, to initiate translation on an mRNA, a ribosome with an initiator methionyl tRNA (Met-tRNA_i_) needs to be positioned at the AUG start codon of the mRNA (2, 3). In most cases, this process is thought to require a basal machinery of 11 initiation factors, the Met-tRNA_i_ and the small (40S) and large (60S) ribosomal subunits. These initiation factors interact either with the mRNA or the ribosomal subunits to recruit and position the ribosomal subunits on the mRNA in a sequential manner (2, 3).

In terms of mRNA interaction, the eIF4F (eukaryotic initiation factor 4F) complex is a heterotrimeric protein complex comprising of eIF4E, eIF4G, and eIF4A that binds the mRNA’s 5′ cap structure (11). eIF4E simultaneously binds both the cap and the scaffold protein eIF4G. eIF4G can also bind to the poly(A) binding protein, Pab1p, raising the possibility that mRNAs can be effectively circularized *via* protein-RNA interactions at both the 5′ and 3′ ends in a so-called “closed loop” structure (12, 13). As well as providing a framework for interactions on the mRNA, eIF4G also provides the critical contact point for the small ribosomal subunit (SSU) directing it to the 5′end of the mRNA (11).

The SSU interacts with a number of translation initiation factors (2, 3). Specifically, eIF2 bound to GTP forms a ternary complex with the Met-tRNA_i_, and this ternary complex, as well as eIF1, eIF5, eIF1A, and the multiprotein complex eIF3, interact with the 40S SSU to form the 43S preinitiation complex (14). Then, by virtue of interaction with eIF4G, this 43S complex makes the key contact with an mRNA near the 5′ end. With the aid of ATP-dependent RNA helicases, such as eIF4A and Ded1p (DDX3X in humans), the 43S complex then scans the mRNA leader for an AUG start codon (15). Once found a series of intermolecular rearrangements, GTP hydrolysis reactions and further translation factor interactions lead to the recruitment of the large ribosomal subunit (2, 3).

In previous studies, we have investigated the relevance of specific translation factors and repressors under both unstressed and stressed conditions in the yeast *Saccharomyces cerevisiae* by performing RNA immunoprecipitations followed by high throughput sequencing (RIP-Seq) (16, 17, 18). We have studied the importance of the closed loop mRNA complex with our data suggesting that, although many mRNAs are bound by Pab1, eIF4E, and eIF4G, some of the most heavily translated mRNAs that produce the most abundant proteins are translated with little apparent interaction with the closed loop, at least in relative terms compared to other groups of mRNA (16, 17). More recent results corroborate these findings and suggest that the closed-loop complex may not be relevant for many mRNAs (19, 20).

As well as selection for translation, a range of other potentially conflicting fates have been described for mRNAs in the cytosol of a eukaryotic cell. For instance, mRNAs are degraded within the cell at varying rates, and one of the most important pathways for the bulk degradation of mRNA is the 5′ to 3′ pathway of mRNA decay (21). Over the lifetime of an mRNA molecule, the mRNA poly(A) tail at the 3′end generally becomes shortened by the action of deadenylases (22). Once shortened beyond a key threshold, the enzymatic removal of the mRNA cap at the 5′end or decapping is stimulated (23). A range of RBPs, such as the cytosolic Sm like (LSm) complex and Pat1 are thought to bind deadenylated transcripts to stimulate the mRNA decapping process (23). RNAs lacking the protection of a 5′ cap structure are rapidly degraded by a cytosolic 5′ to 3′ exoribonuclease of the Xrn family (24). The components involved in this pathway of mRNA degradation can be localized to defined bodies within the cytosol of eukaryotic cells, termed mRNA processing bodies (P-bodies [PBs]) (25). PBs are membraneless biological condensates that, as well as various mRNA decay components, harbor RBPs, translation factors and mRNAs (25). Initial work highlighting the concentration of mRNA decay factors and the accumulation of stabilized decay intermediates at these sites suggested that mRNA degradation might be focussed at these sites (26). However, this suggestion has been challenged by both omics and single molecule studies (27, 28). As a result, it has been suggested that PBs may play a role in mRNA storage either as well as, or instead of, a role in mRNA degradation (29). This highlights the storage of translationally repressed mRNA as another potential fate in the cytosol of an eukaryotic cell. Another heavily studied class of biological condensate that has been thought to play a role in mRNA storage is the stress granule (SG). Under adverse conditions, translationally repressed mRNAs accumulate and can enter SGs. SG constituents partially overlap with PBs, although SGs typically lack the mRNA decay machinery (18). However, a role for SGs in bulk mRNA storage appears less likely based on recent studies where the residency time of mRNA at these sites is quite short (5) and less than 10% of bulk mRNA appears at these loci (30). Nevertheless, the identification and characterization of these different cytoplasmic ribonucleoprotein bodies highlights the extent to which mRNA localization can play a role in mRNA fate. Indeed, we have recently found that specific classes of mRNA encoding glycolytic enzymes or translation factors can be translated at specific sites—core fermentation granules and translation factor mRNA granules, respectively (31, 32, 33).

In order to unravel some of the questions surrounding the specification of mRNA fate, we previously placed mRNAs into different groups based on cross comparing their interaction profiles with some major translation factors: eIF4E, eIF4G, and Pab1p (17). Somewhat surprisingly, we noted that those mRNAs encoding the most highly abundant and efficiently translated proteins did not interact well with eIF4E, eIF4G, or Pab1p, yet paradoxically after stress these mRNAs did interact with these factors (16). Hence, the relevance of these translation factors and the closed loop model to the translation of these mRNAs was questioned. However, in the previous work, we did not study translation initiation factors that interact with the SSU and take part in processes such as scanning and start codon recognition. Equally, we did not address alternative potential mRNAs fates such as mRNA degradation and storage. Therefore, here, we refine our mRNA-centric description of translation selection, by generating further interaction profiles derived from RNA immunoprecipitations of major translation initiation factors such as the γ subunit of eIF2, and the b subunit of eIF3, as well as two RBPs involved in the process of mRNA degradation and storage, Pat1 and Lsm1. By using the resulting profiles, we redefine and extend our mRNA groups based on their integrated interaction properties. These analyses identify distinct modules of similarly controlled mRNAs that likely form the basis for the orchestrated production of proteins with related cellular functions.

## Results and discussion

### Isolation of mRNAs selected for translation or degradation by RIP-seq

**Figure 1:**
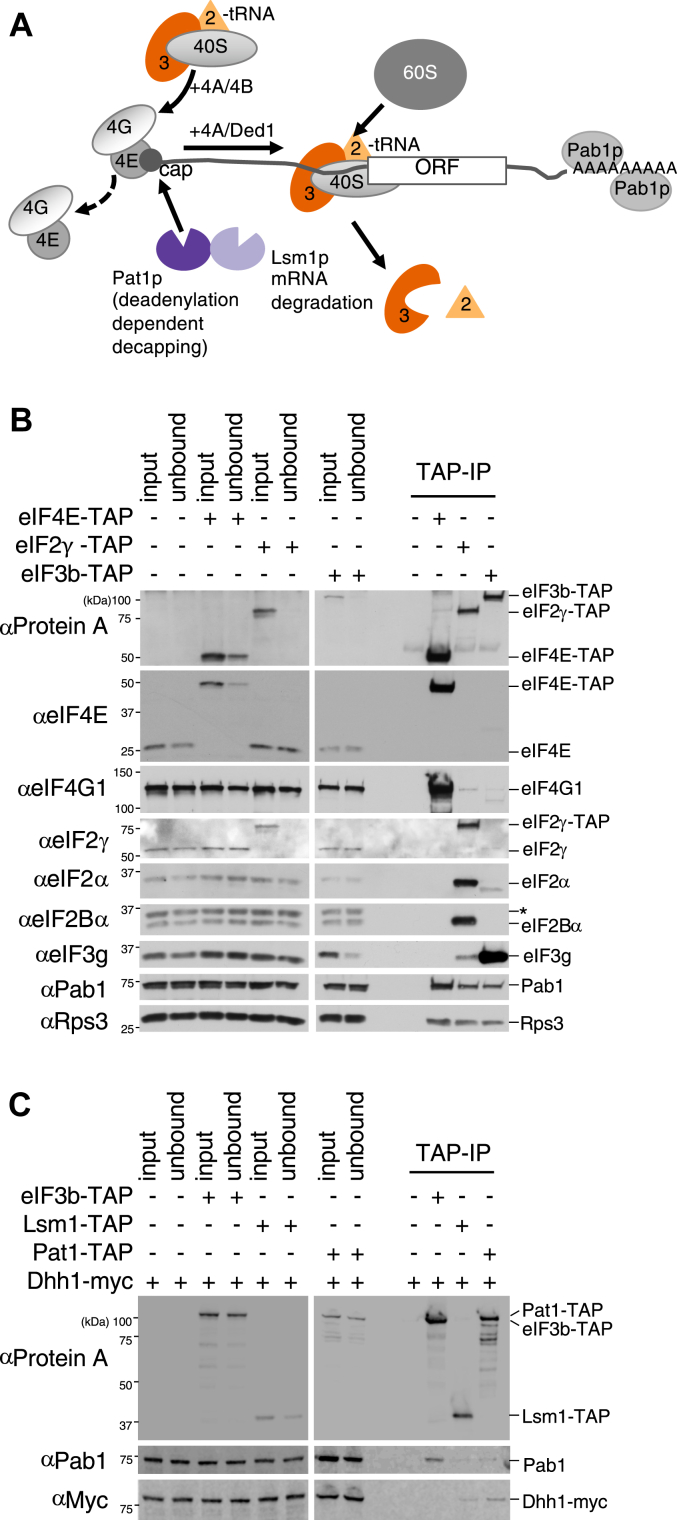
**Specific TAP-tagged translation and mRNA decay factorsbind to a validated set of interacting partners.** Diagrammatic representation of the roles of the translation initiation factors eIF2 and eIF3, and mRNA decay factors Pat1 and Lsm1 in mRNA translation and turnover (*A*). TAP-affinity purifications using whole cell extracts from strains bearing eIF2γ-TAP, eIF3b-TAP, or eIF4E-TAP as a control. Western blots on total (T), unbound (UB) and TAP-immunoprecipitated (IP) samples using antibodies (α) to eIF2 and eIF3 subunits, as well as eIF4G1, Pab1p, and the ribosomal subunit Rps3p (*B*). As (*B*) except Lsm1-TAP, Pat1-TAP, and Prt1-TAP carrying strains were used and anti-myc antibodies were used to detect myc-tagged Dhh1p (*C*). eIF4G1, eukaryotic initiation factor 4F.

Our previous work taking an RIP-seq strategy has characterized mRNA interaction profiles of translation initiation factors, translation repressors, and RNA helicases across the global population of mRNAs (16, 17, 18). This approach has not been previously used to study translation factors that are components of the 43S preinitiation complex, or potentially competing factors involved in mRNA degradation or localization. Therefore, to provide further insights into our mRNA interaction model, we applied our RIP-seq approach to yeast strains where additional translation factors and mRBPs are genomically TAP-tagged (34). We selected the gamma subunit of eIF2 (*GCD11*) and the b subunit of eIF3 (*PRT1*) as examples of translation factors that are required for the ultimate positioning of Met-tRNA_i_ at the start codon (35) (Fig. 1*A*). It is anticipated that these factors would be critical for the vast majority of translation initiation events. In addition, we selected Pat1p and Lsm1p as examples of RBPs associated with mRNA degradation (Fig. 1*A*). Both factors are involved in the 5’ to 3′ mRNA decay pathway where they are thought to interact in the 3′UTR of deadenylated mRNAs, as part of a complex that promotes mRNA decapping (21). Hence both Lsm1 and Pat1 influence the selection of mRNAs for degradation and are present in PBs (18, 21, 25).

The genomic integration of the TAP tag cassette downstream of each ORF for the selected proteins did not affect the global translation profile of the tagged strains as judged by polysome analysis (Fig. S1*A*). In addition, the relative TAP-protein levels for the tagged strains correspond well with previous assessments of protein abundance (Fig. S1, *B* and *C*) (36). Equally, across a series of known protein-protein interactions the TAP-tagged proteins interact appropriately. For instance, the tagged versions of both eIF2γ and eIF3b interact appropriately with other subunits of eIF2 and eIF3 (Fig. 1*B*) (14), and both proteins also interact (likely *via* RNA) with members of the closed loop complex—eIF4G1, eIF4E, and Pab1 as well as a SSU protein marker, Rps3p (Fig. 1*C*). In addition, as predicted from previous studies (37), the TAP-tagged versions of Pat1p and Lsm1p both interact specifically with Dhh1p (Fig. 1*C*).

These strains were therefore taken forward and used to generate RIP-seq datasets to reveal the mRNAs enriched in immunoprecipitations of these factors that occur during active exponential growth. The studies were performed in triplicate, in an identical manner to our previous RIP-seq analyses (16, 17, 18), to enable direct comparison with experiments previously conducted in the laboratory. Raw mapped read counts of these datasets and previous datasets are compiled in Table S1.

### The mRNA decay factors Pat1p and Lsm1p have highly similar profiles of mRNA interaction

The 5′ to 3′ pathway of cytoplasmic mRNA turnover is a continuous process during cell growth and represents a bulk mRNA decay pathway (6). The two selected RBPs, Pat1p and Lsm1p, form part of complicated network of RNA-protein interactions that are important for deadenylation-dependent mRNA decapping by the Dcp1/Dcp2 decapping enzyme (21, 37).

**Figure 2:**
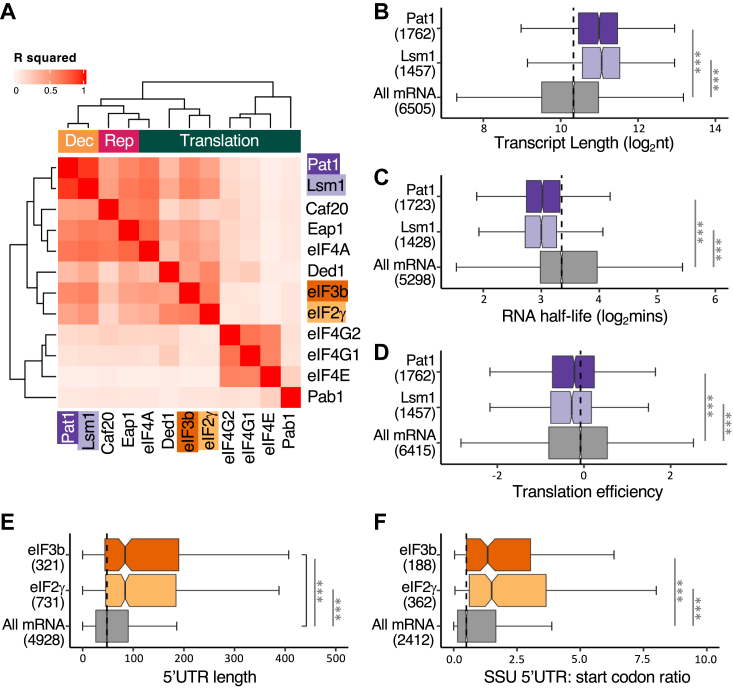
**The properties of mRNAs interacting with the mRNA decay and translation initiation factors are consistent with known degradation and translation mechanisms.** Heatmap depicting the pairwise comparison the four new RIP-seq datasets with each other and other datasets based upon calculated R squared values (*A*). Notched box and whiskers plot depicting the range of transcript lengths (*B*), RNA half-lives (*C*) and translation efficiencies (*D*) for the mRNAs interacting with Pat1p and Lsm1p relative to all mRNAs. Notched box and whiskers plot for 5′UTR lengths (*E*), and small ribosomal footprint positional ratios (*F*) for the mRNAs interacting with eIF2γ and eIF3b relative to all mRNAs. On these plots, the colored boxes depict the extent of the *upper* and *lower* quartiles with the notch representing the 95% confidence interval of the median. Numbers in parentheses indicate how many mRNAs considered in each category. ∗∗∗ adjusted *p*-value < 0.001, ∗∗ adjusted *p*-value < 0.01, ∗ adjusted *p*-value < 0.05. RIP-seq, RNA immunoprecipitation sequencing.

In the past, our RIP-seq data have been most effectively evaluated by calculating the degree of enrichment relative to a total RNA sample from the same yeast culture (17) (Table S2). A comparison of these values for the Lsm1p and Pat1p immunoprecipitation samples reveals that the Lsm1p and Pat1p datasets are strongly correlated with one another. For instance, in a pairwise analysis of the log_2_ (immunoprecipitation (IP)/total) values for matched mRNAs across our new and all our previous RIP-seq datasets, we find that the Lsm1p *versus* Pat1p comparison generates the highest pairwise correspondence (R^2^ = 0.91) across all the RIP-seq experiments we have performed (Fig. 2*A*). Since Lsm1p and Pat1p interact with each other (37), play roles in the same phases of the mRNA decay pathway (21), and are both components of PBs (26), the similarity in their RIP-seq data confirms the robustness and relevance of our approach.

Unsurprisingly, given the high overlap between the Pat1p and Lsm1p profiles, these datasets also correlate with other mRNA properties revealed in published datasets (18, 38, 39). For instance, mRNAs which interact robustly with Lsm1p and Pat1p are longer than the genome average (Fig. 2*B*), have shorter half-lives (Fig. 2*C*) and have lower ribosome occupancies (typically termed “translation efficiency”) (Fig. 2*D*). Indeed, the repression of translation initiation appears to represent a prerequisite for the entry of mRNA to PBs (40) and correlates with mRNA degradation (41). Hence, the fact that the Pat1p/Lsm1p RIP-seq enrichment profiles correlate well with those of eIF4E-binding proteins Caf20p and Eap1p (Fig. 2*A*), which are translation repressors, is also consistent with these previous findings. Additional correlations are observed in comparisons with mRNAs bound by the decapping complex members, Dcp1 in mRNA PBs and Pbp1 in SGs (18) (Fig. S2, *A* and *B*) (*p*-values < 0.0001). Collectively, these results point to the Pat1p and Lsm1p RIP-seq profiles acting as a measure of the predisposition of mRNAs toward a degradation/storage fate. Consequently, they add a distinct and novel proxy measure to the overall profile of mRNA selection for translation bringing in different aspects of the mRNA life cycle.

### eIF2 and eIF3 interactions correlate with 43S ribosomal scanning

In previous studies, we have characterized the mRNA interaction profiles of those translation initiation factors known to interact with the mRNA 5′ cap and 3′ poly(A) tail (16, 17), but we did not examine those components that interact with the 40S ribosomal subunit directing it to the mRNA. To explore the mRNA interaction profile of such factors, we performed RIP-seq on both eIF2γ (Gcd11) and eIF3b (Prt1), which are subunits of eIF2 and eIF3 respectively. These translation factors play multiple roles in the translation process (14). As well as forming part of the 43S preinitiation complex that interacts with the eIF4F complex bound to the mRNA 5′ cap, both eIF3 and eIF2 play key roles at downstream stages in translation initiation such as mRNA 5′UTR scanning and start codon recognition (14). For eIF3, there is also evidence that this factor remains bound to at least some mRNAs during postinitiation phases of translation to impact on early translation elongation, termination, and ribosome recycling (42, 43).

The eIF2 and eIF3 RIP-seq datasets share highly similar interaction profiles (R^2^ value of 0.62) (Fig. 2*A*), as might be expected, since both factors interact with the SSU, and play roles in mRNA scanning and AUG recognition (14). A cross-comparison with our previous RIP-seq data (17, 18) highlights that the eIF2 and eIF3 profiles are most similar to the profiles of the ATP-dependent RNA helicases, Ded1p (R^2^ values of 0.58 and 0.43 for eIF2 and eIF3, respectively) and eIF4A (R^2^ values of 0.36 and 0.46 for eIF2 and eIF3, respectively) (Fig. 2*A*). This is intriguing since Ded1p is thought to unwind structured mRNA 5′UTRs to facilitate the scanning process whereby the 43S ribosomal complex sequentially checks the mRNA to locate the start codon, and eIF4A is likely involved in 43S recruitment to the mRNA for most mRNAs (44). It is likely therefore that the correlation between these datasets stems from the involvement of both Ded1/eIF4A and eIF2/eIF3 in the 43S ribosomal complex scanning and AUG recognition process on the mRNA.

Some of the physical properties of the most robustly eIF2 and eIF3 enriched mRNAs also support a view that these factors interact with mRNAs enriched in scanning 43S ribosomes. For instance, on average these mRNAs have significantly longer 5′UTRs, a trait that might be anticipated for mRNAs accumulating 43S ribosomal components (Fig. 2*E*). Ribosome profiling techniques have also been used to evaluate the level and position of SSU interactions with mRNAs (45). A particularly informative evaluation is the ratio of SSU interactions across the 5′UTR *versus* those directly over the start codon (45). For the mRNAs most robustly associated with eIF2 and eIF3 this ratio is significantly enhanced suggesting that these mRNAs accumulate SSUs over the 5′UTR (Fig. 2*F*). Once again, this comparison is consistent with the eIF2 and eIF3 profiles enriching mRNAs where the scanning 43S ribosomal complex accumulates. Therefore, these data capture a distinct aspect of translational regulation to those RIP-seq datasets that we have interrogated previously.

Interestingly, the time taken for initiation events *in vivo* is estimated to vary by around two orders of magnitude from the fastest mRNAs initiating every few seconds to those taking over 200 s with a median of 40 s (46). It appears from the above discussion that our RIP-seq approach enriches mRNAs where initiation is slower. Although our IP takes longer than the time for initiation (15 min), it is performed at 4 °C (47), which likely slows exchange of these dynamic RNA-protein interactions.

### Ded1 interaction profile correlates precisely with the degree to which mRNAs are affected by *DED1* mutations

In order to evaluate the validity of our IP strategy, we compared our data from the Ded1p mRNA interaction profile with previous datasets. For instance, a comprehensive analysis of the impact of *DED1* mutations on the global density of ribosomes across mRNAs has been used to define a directory of mRNAs that are Ded1-dependent (44). A comparison of these Ded1-dependent mRNAs with our Ded1 RIP-seq dataset reveals a remarkable overlap (Fig. S2*C*) (*p*-value < 0.0001). This correlation is especially telling given that two completely different technical strategies have been used in different yeast strains to evaluate those mRNAs where Ded1 is important. The correlation cross-validates both of these complementary technical approaches and provides independent corroboration of the results from our RIP-seq approach.

### The eIF4A mRNA interaction profile is highly similar to that of the eIF4E binding proteins

A final point worth noting from the pairwise comparisons of the RIP-seq datasets (Fig. 2*A*) is that the eIF4A profile is highly similar to the profiles of the two yeast eIF4E binding proteins (4E-BPs), Eap1p and Caf20p (R^2^ values of 0.73 and 0.65, respectively). Eap1p and Caf20p have been thought of as translation repressors acting to curtail translation initiation through interactions with eIF4E and/or *via* interactions with the ribosome (48, 49, 50, 51). Indeed, both proteins interact with eIF4E to inhibit translation *in vitro* (52, 53), and deletion of the *CAF20* gene suppresses, whereas *CAF20* overexpression exacerbates certain translation factor mutations, including mutations in eIF4A (*tif1-1*) (54). However, a recent *in vitro* study on cap-dependent translation using extracts from eIF4E mutant strains found that addition of a Caf20-eIF4E complex stimulated translation more effectively than adding eIF4E alone, raising the possibility that the 4E-BPs can also activate protein synthesis in certain contexts (55).

Experiments investigating the impact of eIF4A mutations suggest that eIF4A is required for the translation of most if not all mRNAs (44). In keeping with this, regulation at the level of eIF4A following glucose depletion in yeast leads to a particularly widespread overhaul of yeast translation (56, 57). eIF4A is also one of the most abundant translation initiation factor with levels exceeding many ribosomal proteins (58). Therefore, it seems likely that eIF4A interacts with most mRNAs at some point in their life cycle. The correlation of the eIF4A mRNA interaction profile with that of the 4E-BPs is suggestive that eIF4A may accumulate on translationally repressed mRNAs. It is possible that the accumulation of eIF4A on these mRNAs could facilitate an exchange of the 4E-BP molecule for eIF4G during renewed translation initiation. Alternatively, eIF4A has been described to play roles in the condensation of untranslated mRNAs to form SGs, so the overlap with the 4E-BPs may stem from this modulation of RNA containing condensates (59).

### Seven unique mRNA cohorts identified *via* differential engagement with the 43S complex, the closed-loop complex and the mRNA decay pathway

**Figure 3:**
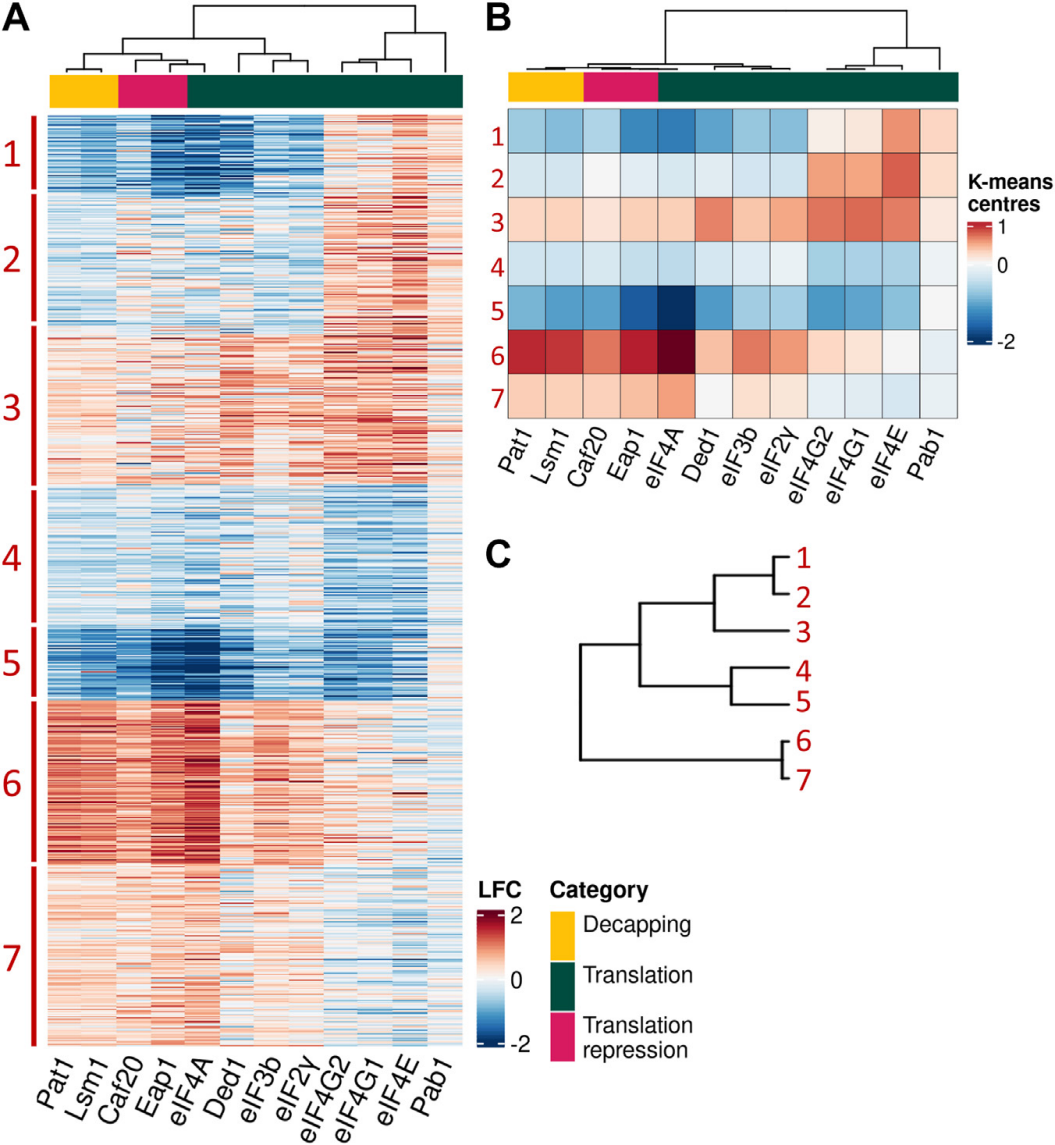
**Cross-comparison of RIP-seq profiles from 12 translation factors or RBPs.** Heatmap of LFC values of genes enriched or depleted in at least one RIP-seq experiment. Experiments ordered by similarity, differentially represented RNAs ordered in k-means clusters (*A*). Heatmap of the k-means centers of the LFCs in each cluster for each target protein (*B*). Dendrogram representing the relationship between the seven clusters (*C*). LFC, log2 fold changes; RBP, RNA binding proteins; RIP-seq, RNA immunoprecipitation sequencing.

While an analysis of the pairwise correlation between RIP-seq datasets as well as the properties of enriched mRNAs can provide useful information, it is also clear that significant extra insight can be obtained by integrating the data across multiple RIP-seq experiments (16, 17). Therefore, to generate a more holistic view of the RNA interaction profiles of our new RIP-seq datasets, they were integrated with our previously published datasets (17, 18). This integration has meant that a much larger fraction of the total transcriptome is now considered relative to our previous analyses (16, 17). In line with our previous work, our analysis is restricted to those transcripts displaying a significant (false discovery rate (FDR) <0.01) enrichment (or underrepresentation) according to EdgeR’s generalized linear model in at least one of the IPs, as well as to transcripts with greater than 20 reads in each of the pertinent total extract samples. Overall, a combined total of 5050 mRNAs are now considered. This represents nearly 90% of the Saccharomyces Genome Database-annotated mRNAs, which are subdivided into seven clusters on the heatmap analysis (Fig. 3, *A* and *B*). Each cluster possesses a unique enrichment profile across the RIP-seq datasets (Table S3), although clearly from the dendrogram some clusters are more similar than others. The data broadly partition the yeast transcriptome into three major groups: clusters 1, 2, and 3; clusters 4 and 5; and clusters 6 and 7 (Fig. 3*C*), so these are discussed separately over the next sections.

### mRNAs enriched with translation repressors, the mRNA decay machinery and RNA helicases are poorly translated

**Figure 4:**
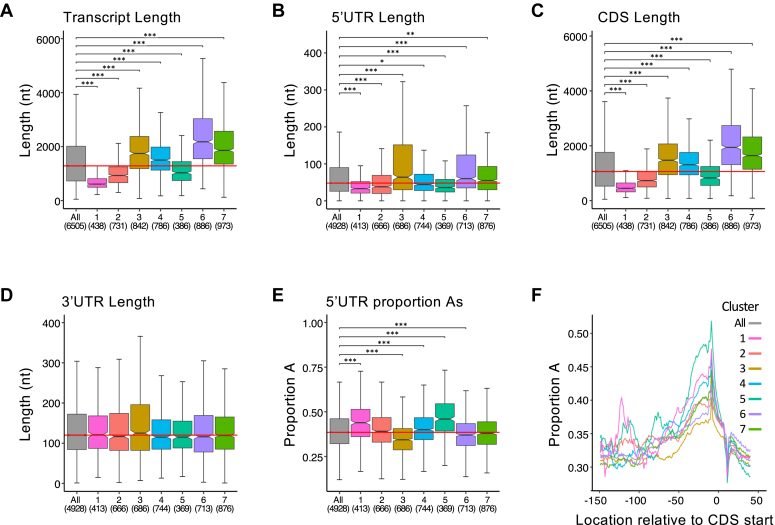
**The physical properties of mRNAs within the seven clusters.** Notched box and whiskers plots detailing the properties of mRNAs within each of the seven clusters compared to all mRNA. Numbers in parentheses are the number of mRNAs from each cluster. Properties considered include the following: transcript length (*A*), 5′ UTR length (*B*), coding sequence (CDS) length (*C*), 3′ UTR length (*D*) and proportion of adenosine residues in the 5′ UTR (*E*). Numbers in parentheses indicate how many mRNAs were considered. Plot detailing the relative enrichment for adenosine residues over the 5′UTR for each cluster (*F*). Box and whisker definition as described in legend to Figure 2.

Cluster 6 is enriched with the 4E-BP translation repressors (Caf20p and Eap1p), with the mRNAs decay factors (Lsm1p and Pat1p), and with the RNA helicase eIF4A, whereas for the other translation factors, levels of enrichment are much less pronounced (Fig. 3, *A* and *B*). Cluster 7 seems to represent a milder version of cluster 6 in terms of the enrichments observed (Fig. 3, *A* and *B*). Both clusters are comprised of relatively long mRNAs (Fig. 4*A*), where the increased length relative to other clusters stems largely from the length of the coding sequence rather than 5′ or 3′ UTR lengths (Fig. 4, *B*–*D*).

**Figure 5:**
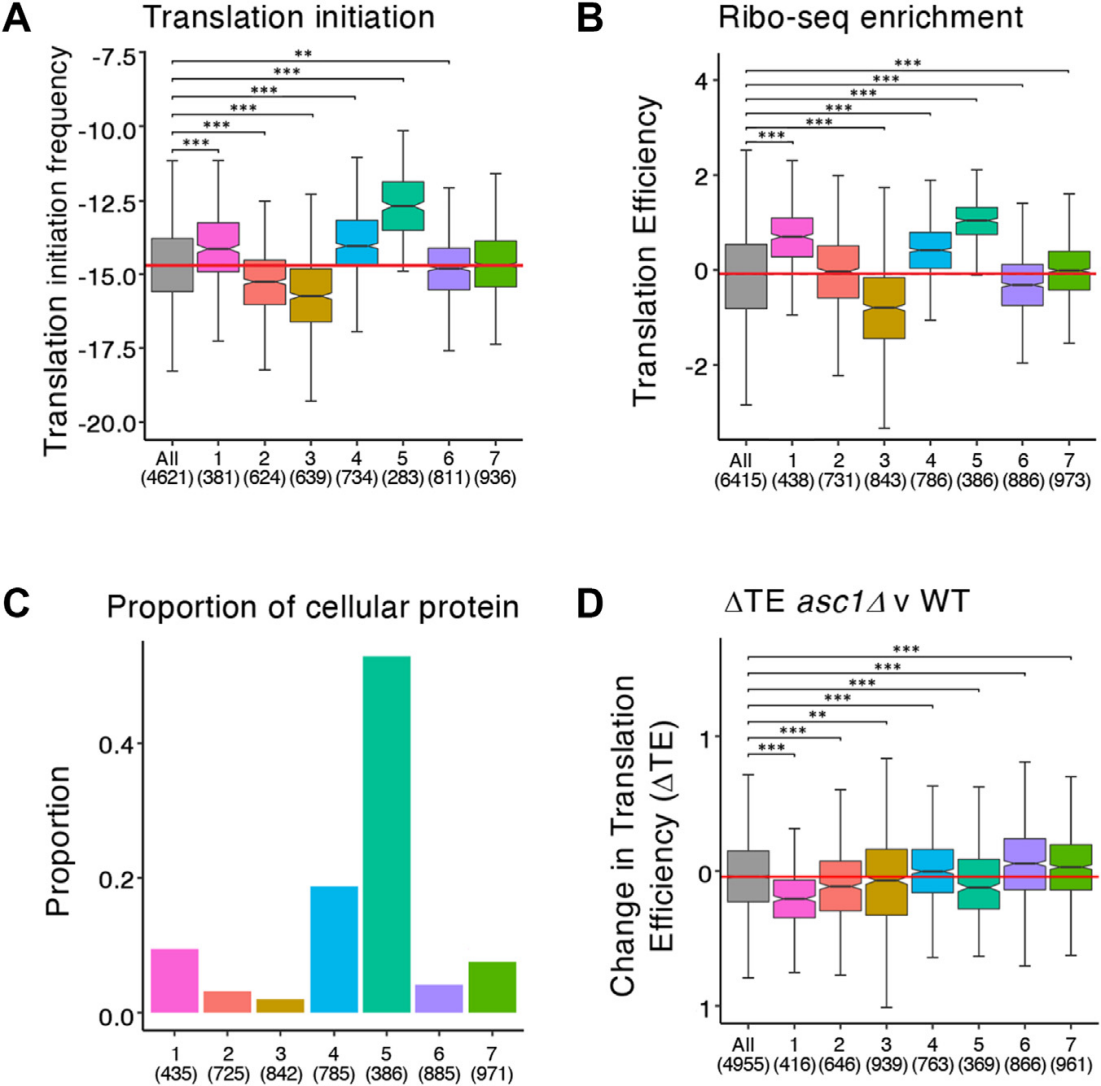
**Translational properties of the mRNAs within the seven clusters.** Notched box and whiskers plots of the translation initiation frequencies of mRNAs (60) in each cluster compared with all mRNA (*A*). Translational efficiency of mRNAs (18) in each cluster compared with all mRNA (*B*). Proportion of cellular protein encoded by mRNAs (61) in each cluster from paxDB (*C*). A notched box and whiskers plot for the change in translation efficiency observed in an *ASC1* mutant relative to the parent strain for mRNAs in each of the seven clusters reactive to all mRNAs (71) (*D*). Numbers in parentheses indicate how many mRNAs were considered. Box and whisker definition as described in legend to Figure 2.

**Figure 6:**
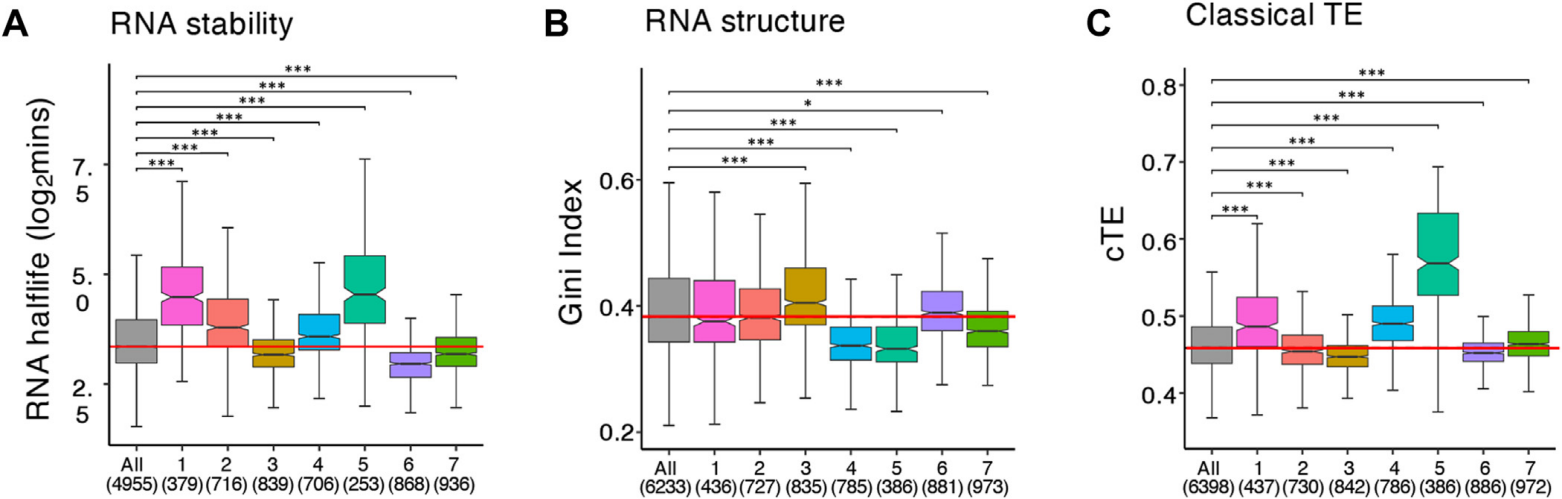
**Stability, structure, and codon preferences of the mRNAs within the seven clusters.** Box and whiskers plots of RNA stability (39) of mRNAs in each cluster relative to all mRNAs (*A*), RNA structure (73) in each cluster relative to all mRNAs (*B*) and the classical TE calculated (78, 79) using the tRNA adaptation index and codon usage for the mRNAs in each cluster relative to all mRNAs (*C*). Numbers in parentheses indicate how many mRNAs considered for each cluster. Box and whisker definition as described in legend to Figure 2. TE, translational efficiency.

The translation efficiencies and levels of translation initiation on these mRNAs, as taken from ribosome profiling datasets (18, 60, 61), follow the global average, with cluster 6 mRNAs (those enriched with repressors and RNA decay factors) generally being slightly less well engaged with ribosomes than cluster 7 (Fig. 5, *A*–*C*). In addition, cluster 6 mRNAs have shorter half-lives (39) than the mRNAs in other clusters (Fig. 6*A*), and Cluster 7 mRNAs have less structure (Fig. 6*B*). These correlations suggest that clusters 6 and 7 comprise mRNAs where translation is not efficient and other fates for the mRNAs such as translation repression and mRNA degradation may be important in regulating expression of the mRNAs involved.

A gene ontology (GO) analysis of the molecular and biological functions of the proteins encoded by the cluster 6 and 7 mRNAs highlights a range of quite diverse categories. For instance, cellular bud neck, polarized growth, serine threonine kinase activity, and transcription regulators are all highlighted (Fig. S3, *A*–*C*). Since most of these processes and activities are regulatory in nature, the level of translation and hence protein produced from these mRNAs might be expected to be low. Indeed, some mRNAs in these clusters express proteins with known oscillatory dynamics such as *CLB2*, *CLN1*, *CDC15*, and *WHI5* (62). From the data presented here, it seems likely that part of this control of protein levels for the cluster 6 and 7 mRNAs might involve the repression of mRNA translation and degradation *via* interactions with 4E-BPs and the mRNA decay machinery.

### mRNAs interacting with the closed loop components have variable translation rates

Clusters 1, 2, and 3 contain mRNAs that are strongly enriched with the translation factors eIF4E, eIF4G, and Pab1p and would therefore have the potential to form a closed loop messenger RNP complex. As well as the closed loop factors, cluster 3 mRNAs are enriched with most other factors included in the analysis. In contrast, cluster 1 and 2 mRNAs are depleted from the immunoprecipitations of most other factors (Fig. 3, *A* and *B*), with cluster 2 mRNAs representing a milder version of cluster 1.

Interestingly, the mRNAs that are present in these three clusters have diverse physical properties. For instance, cluster 1 and 2 mRNAs are shorter, whereas cluster 3 mRNAs are longer than the norm (Fig. 4*A*). The shorter transcripts in clusters 1 and 2 arise from both shorter 5′UTRs and shorter coding sequences (CDS) without significant differences in the 3′UTR length (Fig. 4, *B*–*D*). In contrast, mRNAs in cluster 3 have longer transcripts with both longer 5′UTRs and CDS but again little significant difference in 3′UTR length (Fig. 4, *B*–*D*).

When the translation level of the mRNAs found in clusters 1, 2, and 3 is cross-compared, cluster 1 mRNAs are revealed as the most heavily translated and encode the most abundant proteins (Fig. 5, *A*–*C*), cluster 2 mRNAs are a milder version of cluster 1 and cluster 3 mRNAs are relatively poorly translated. For cluster 1 mRNAs, a GO analysis reveals that ribosome, cytoplasmic translation, and mitochondrial proteins are prominent enriched GO categories (Fig. S3). The high level translation of cluster 1 mRNAs represents an apparent paradox, since mRNAs that are depleted of key translation factors (such as eIF2 and eIF3, and the helicases eIF4A and Ded1p) form the basis of this heavily translated cluster. One possibility is that given the shorter than average length of the 5′UTR for these mRNAs, the process by which the 43S complex locates the mRNA start codon is very efficient. This would mean that at steady state neither the 43S components such as eIF2 and eIF3 nor the mRNA helicases accumulate on these mRNAs. As discussed later for cluster 5, it is also possible that the higher translation of cluster 1 mRNAs relates to the adenosine (A) content in the 5′UTR of these mRNAs, especially where the high A content lies just upstream of the start codon.

Cluster 3 differs from the others in that its member mRNAs are enriched with every factor analyzed (Fig. 3, *A* and *B*). This is possibly because it contains long transcripts with long 5′UTRs and long coding sequences (Fig. 4, *A*–*C*). Cluster 3 mRNAs are also the least frequently initiated mRNAs (Fig. 4*A*) with the lowest density of ribosomes (Fig. 4*B*) and contributes the least to the cellular proteome (Fig. 4*C*). The transcripts have lower than average half-lives and the highest secondary structure scores (Fig. 6, *A* and *B*). These properties combine to imply that these mRNAs encode proteins needed at low amounts and so are not optimized for efficient translation.

Overall, it is clear from the profiles and properties of the mRNA in clusters 1, 2, and 3 that enrichment with the mRNA selection machinery (eIF4E, 4G, and Pab1), which represents the defining feature of these clusters, is not necessarily a predictor of high levels of translation initiation. Instead, there are complex interactions with other RBPs and translation factors, which combined with the key properties of the mRNAs themselves such as the length of the 5′UTR likely account for translation levels of mRNAs in these clusters.

### The most heavily translated mRNAs interact poorly with the translation initiation machinery

Even though cluster 1 mRNAs are translated more heavily than most, clusters 4 and 5 are responsible for producing a major part (>80%) of the protein molecules in the cell (Fig. 5*C*). Of all 7 clusters, cluster 5 is the smallest cluster containing 386 mRNAs, yet it is the source of over half of the cellular protein content (Fig. 5*C*). Correspondingly, the mRNAs found in this cluster have a high translational efficiency (Fig. 5, *A* and *B*), and although they represent the most heavily translated mRNAs in the cell, these mRNAs are the most under enriched with the closed loop machinery. Indeed, the mRNAs in cluster 4 and 5 are under enriched in immunoprecipitations of most factors tested, except Pab1 (Fig. 3*A*).

The enrichment with Pab1 combined with the underrepresentation of other factors is an intriguing observation given the other properties of these mRNAs. More specifically, the cluster 5 mRNAs are short, with especially short 5′UTRs (Fig. 4, *A* and *B*) that are enriched for adenosine residues (Fig. 4*E*) especially in the vicinity just upstream of the start codon (Fig. 4*F*). This tendency is also apparent for the cluster 1 mRNAs described above, which are also short and efficiently translated (Fig. 4*A*). A-rich sequences immediately upstream of the AUG sequence have previously been noted on highly expressed mRNAs and are known to promote highly efficient translation in yeast (63, 64). Indeed, it has also been suggested that A-rich sequences near the AUG can act to recruit ribosomes in a mechanism where Pab1 interaction results in the cap-independent recruitment of eIF4G (65). Such a mechanism has parallels with the targeting of poly(A) binding proteins to the 5′UTR in plant internal ribosome entry site-dependent translation initiation (66) and to an internal ribosome entry site in the 5′ leader of an avian herpesvirus (67). Direct targeting of the 40S ribosomal subunit to the start codon *via* Pab1 would obviate the necessity for scanning perhaps explaining why the cluster 5 (and cluster 1) mRNAs are not enriched with eIF2/eIF3 or the RNA helicases. Alternatively, the adenosines immediately upstream of the start codon could promote more efficient start codon recognition on the mRNA. This might decrease the dwell time of scanning ribosomes hence reduce the level of eIF2/3 and the RNA helicases associated with the mRNA. Another not mutually exclusive possibility is that the translation of cluster 5 mRNAs is dealt with differently within the cell. For instance, this cluster contains most of the glycolytic and many translation factor/ribosomal protein mRNAs with “carbohydrate metabolic process” and “cytoplasmic translation” representing particularly prominent GO terms (Fig. S3*A*). Our recent studies suggest that both of these classes of mRNA can be translated within localized multi-mRNA foci in actively growing cells, termed translation factories (31, 33). The rules governing ribosome recruitment within such factories are not known, and may differ to those elsewhere in the cell given the potentially altered concentration of the translation machinery and the divergent physical environment that likely accompanies such factories. The A-rich sequences near the start codon may well form part of this distinct translation mechanics.

Another intriguing observation concerning the translation of mRNAs across the 7 clusters relates to a potential role for the yeast homolog of RACK1, Asc1p. Asc1p is part of the 40S ribosome and interacts near the mRNA exit channel, where it has roles in ribosome quality control pathways (68, 69). Asc1p/RACK1 also has a range of signaling functions possibly targeting signaling pathways toward the ribosome (70). Previous work has shown that Asc1p can play key roles in translation control where it is important in the efficient translation of short mRNAs that interact well with the translation initiation factors eIF4E and eIF4G and in the translation of mRNAs during heat stress (71, 72). From our new clustering analysis, both cluster 1 and 5 mRNAs are especially sensitive to deletion of the *ASC1* gene, since these clusters exhibit the largest changes in translation efficiency (Fig. 5*D*). This suggests that Asc1 may be important in the translation of mRNAs with short 5′UTRs. Such mRNAs either interact with eIF4E/4G (cluster 1) or they interact poorly with most factors barring Pab1 (cluster 5). Therefore, deletion of this ribosomal protein appears to affect the translation of only certain mRNAs highlighting potential ribosome specificities for the heavily translated mRNAs that form the core of clusters 1 and 5.

### The relationship between mRNA structure, stability, and translation

The functional grouping of mRNAs based on their interaction profiles has revealed both physical and functional similarities for mRNAs with similar binding properties. Analysis of the stability and structure using published datasets (39, 73) for the mRNAs present across the 7 clusters provides further insight (Fig. 6, *A* and *B*). For instance, it is intriguing that the translational activity of the various clusters almost precisely mirrors RNA half-life (*cf* Figs. 6*A* with 5*B*). Clusters with heavily translated mRNAs are generally more stable, whereas poorly translated clusters contain generally less stable mRNAs. The relationship between the various steps in translation and mRNA decay are highly complex (74). For instance at the initiation stage, translation can be thought of as competing with degradation processes, such that mutations and conditions inhibiting translation initiation lead to increased mRNA degradation (41, 75). Whereas, a recent focus in the field has been the correlation between codon optimality, translation elongation, and mRNA stability (76, 77). Overall, the correlations above fit well with our data where for those clusters with heavily translated, more stable transcripts, the mRNAs have high translation initiation rates, carry optimal codons and have higher rates of translation elongation as evidenced by their higher classical translation efficiency (*cf* Fig. 5*A* with Fig. 6*C*), an expression which takes into account the tRNA adaptation index and codon usage (78, 79).

The degradation rates of mRNA are also heavily influenced by RNA structure (80). Therefore, our seven clusters were assessed in terms of the structure of their constituent mRNAs using a dataset where structure was assessed using a dimethyl sulfate (DMS)-seq approach, which condenses down to a single value or Gini index score. Here, the higher the Gini index, the more likely an mRNA is to contain structured regions (73) (Fig. 6*B*). Interestingly, clusters 4 and 5 are significantly less structured than average, yet more stable and more heavily translated (*cf* Fig. 6*B* with Figs. 6*A* and 5*A*). In contrast, for cluster 1, which is also heavily translated and highly stable (Figs. 5*A* and 6*A*), the overall propensity to be highly structured is not significantly different to the overall mean (Fig. 6*B*). Therefore, the relationship between RNA structure and mRNA stability is complex and does not always correlate with translation. Our results highlight that at least part of this complexity may relate to the degree to which mRNAs interact with the translation initiation factors associated with the closed loop complex.

### A diverse cohort of RBPs account for the differences between specific clusters of mRNAs

The cohort of RBPs that are associated with an mRNA are thought to be integral to the biological fate of that mRNA. For instance, the RNA regulon model of the mRNA fate posits that the complex configurations of RBPs found on mRNAs are similar for functionally related mRNAs and, thus, there is considerable scope for coordinated regulation (81, 82, 83, 84).

**Figure 7:**
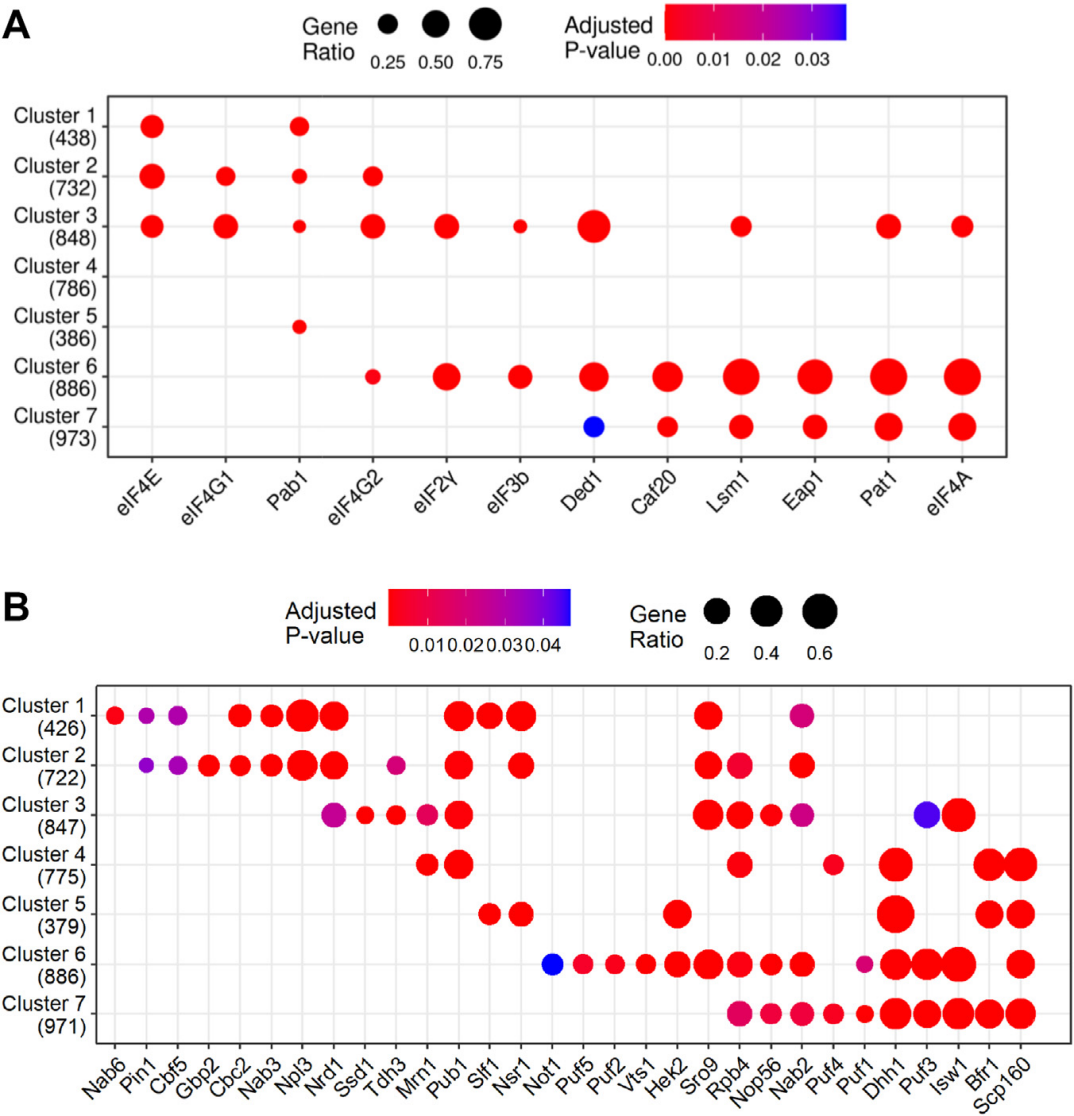
**Cross-referencing of the cohorts of mRNAs that bind to specific RBPs from a range of studies relative to mRNAs within the 7 clusters.** Enrichment level (spot size) and the adjusted *p*-value (*color*) plotted for the overlap between mRNAs present in each of the seven clusters and mRNAs enriched with the listed proteins using (*A*) the data from our own studies (17, 18) highlighting the distinctions in RBP and translation factor/RBP enrichment levels, and (*B*) the data from other studies using a host of RBP mRNA binding lists (85, 86, 87, 88, 89, 90, 91). Numbers in parentheses show how many mRNAs considered for each cluster. RBP, RNA binding proteins.

There have been many previous studies in *S. cerevisiae* investigating which RNAs are bound by specific RBPs (85, 86, 87, 88, 89, 90, 91). Therefore, we cross-referenced the cohorts of mRNAs that bind to specific RBPs from the literature with the 7 clusters that we have identified in this work (Fig. 7). Reassuringly, cross-referencing with our own data used to generate the clusters shows that the enrichment pattern of RBPs relative to clusters varies for each cluster (Fig. 7*A*). Furthermore, the cross-referencing to other RBP datasets reveals a variation in pattern that is similar to the dendrogram depicting how well the clusters relate to one another (*cf* Fig. 7*B* with Fig. 3*C*). Hence, while no single RBP is discriminatory for a certain cluster, the pattern of RBP correlations allows cluster differentiation. The one RBP shared by only the two most heavily translated clusters, clusters 1 and 5, is Slf1p. This La motif containing RBP was recently implicated as playing a role maintaining elongating ribosomes in the correct reading frame rather than in ribosome recruitment (92). Therefore, it is likely that the RBPs from these datasets influence far more than simply mRNA selection for translation but are involved in governing the wider fate of the mRNA. Overall, this analysis suggests that the differential fate of mRNAs correlates with the RBP interaction profile of that mRNA, and that this is coordinated across most of the transcriptome.

## Conclusions

Here, we describe a detailed global analysis of the patterns that exist in the mRNA interactomes of diverse RBPs. The factors selected have wide-ranging roles in translation initiation, mRNA decay and mRNA storage, and the data suggest that their mRNA interactions are coordinated in a manner that correlates with the physical and functional properties of the mRNAs in question. These results are consistent with a hypothesis for the posttranscriptional control of gene expression that was first posited over 20 years ago: namely the RNA regulon model (81, 82, 83, 84). Previous work supporting this model has highlighted how posttranscriptional control of individual pathways or processes is coordinated by RBPs or regulatory RNAs (83, 93, 94, 95). Here, we go further by showing that most of the yeast transcriptome is orchestrated into broad yet distinct posttranscriptional regulons that likely interact with a defined cohort of RBPs to coordinate mRNA fate and function.

## Experimental procedures

### Yeast strains and growth conditions

All yeast strains were grown in synthetic complete dextrose media lacking histidine (ForMedium) at 30 °C. BY4741 TAP-tagged strains of Gcd11p, Lsm1p, Pat1p, and Prt1p were obtained from Thermo Fisher Scientific Open Biosystems. A Cdc33-TAP strain was produced for a previous study as was a BY4741 *HIS3* strain (17) that was used as a control for the immunoprecipitations. Pat1-TAP Dhh1-myc and Lsm1-TAP Dhh1-myc strains Myc-tagged strains were constructed and verified using PCR-based strategies in yeast strain BY4741 *HIS3* (96).

### Ribosome cosedimentation analysis

Polyribosome analysis was performed as previously described (97). Briefly, yeast cells were grown in synthetic complete dextrose (SCD)-His to an *A*_600_ of 0.6 to 0.7 then cycloheximide was added to a final concentration of 0.1 mg/ml and cultures were rapidly chilled in an ice/water bath. Cells were harvested by centrifugation, washed with polysome lysis buffer, and lysed into polysome lysis buffer containing cycloheximide using acid washed glass beads (all at <4 ^°^C). 2.5 *A*_260_ of the cleared yeast lysate was loaded onto a 15 to 50% w/v sucrose gradient which was centrifuged for 2.5 h at 40,000 rpm using a Beckman SW41 rotor at 4 °C. Polysome traces were produced by continuously measuring the *A*_254_ of the sucrose gradient from the top of the gradient as 60% sucrose was added to the bottom using a UA-6 UV/Vis detector and chart recorder (Teledyne ISCO).

### Immunoprecipitation of TAP-tagged proteins

Immunoprecipitations of TAP-tagged proteins were performed as previously described (47). Briefly, cells were grown to an *A*_600_ of 0.6 to 0.7, frozen in liquid nitrogen, and lysed using a Spex freezer Mill 6870 into Lola140: 20 mM Tris–HCl (pH 8), 140 mM NaCl, 1 mM MgCl_2_, 0.5% NP40, 0.5 mM DTT, 1 mM PMSF, EDTA free protease inhibitor cocktail tablet (Roche Diagnostics), 100 μM NaV_3_O_4_, 5 mM NaF, and 40 units/ml RNasin (Promega). Cell lysates were applied to Rabbit IgG coupled Tosyl-activated Dynabeads M-280 magnetic beads for 15 min at 4 °C with rotation. Pellets were washed five times in Lola140 containing 10 U/ml RNAsin.

### RIP-seq analysis

RNA preparation, processing, and sequencing from total and immunoprecipitated samples was performed as described previously (17). Briefly, RNA was isolated and purified using TRIzol LS reagent (Invitrogen) from input yeast lysate or immunoprecipated TAP-tagged protein, quantified using a Nanodrop 8000 spectrophotometer (Thermo Fisher Scientific) and rRNA depleted using RiboMinus Concentration Modules (Life Technologies). rRNA depletion was confirmed using a Bioanalyzer (Agilent Technologies). rRNA depleted total and immunoprecipitated RNA (0.1–4 μg) was used as input material which was fragmented using divalent cations under elevated temperature and then reverse transcribed into first strand complementary DNA (cDNA) using random primers. Second strand cDNA was then synthesized using DNA polymerase I and RNase H. Following a single “A” base addition, adapters were ligated to the cDNA fragments, and the products then purified and enriched by PCR to create the final cDNA library. Adapter indices were used to multiplex libraries, which were pooled prior to cluster generation using a cBot instrument. The loaded flow-cell was then paired-end sequenced (76 + 76 cycles, plus indices) on an Illumina HiSeq4000 instrument. Finally, the output data were demultiplexed (allowing one mismatch) and BCL-to-Fastq conversion performed using Illumina’s bcl2fastq software, version 2.17.1.14. Fastq files were mapped to the *S. cerevisiae* genome (sacCer3 assembly (R64-1-1) obtained from Ensembl) with Bowtie 2 (98). Reads were processed with Samtools (99) and assigned to genes using HTSeq-count (100) using the corresponding R64-1-1 gtf annotation of the *S. cerevisiae* genome. These raw counts were then processed by EdgeR (101) to calculate significant enrichments of transcripts in the IP samples relative to TAP-tag whole transcriptome extracts, using the generalized linear model functionality with a paired statistical design. Enriched or depleted transcripts were those with an FDR of less than 0.01.

### Data analysis

The four new RIP-seq experiments reported here were combined with our previous published RIP-seq datasets that were generated in the same manner. These data are from immunoprecipitations of the four members of the closed loop complex, two eIF4E binding proteins (17) and the RNA helicases eIF4A and Ded1 (18). R^2^ correlation between different RIP-seq datasets was calculated using the complete set of RIP-seq log_2_ fold change (LFC) values. The Pearson correlation values were used to calculate a dissimilarity matrix between the experiments as distance = 1-R. Hierarchical clustering was performed using the R package hclust on the dissimilarity matrix. The plots detailing the resultant R^2^ value and the relationship between RIP-seq experiments were produced using ComplexHeatmap (102). Overlap with previous enriched transcript lists were tested for statistical significance with the hypergeometric test for paired comparisons and 1,000,000 random draw simulations for three-way overlap.

Cluster analysis was performed with a combined list of all RNAs that were significantly enriched or depleted (FDR less than 0.01) in at least one of the 12 RIP-seq datasets, resulting in a list of 5050 RNAs. The R package pheatmap was used to cluster genes based on their correlation of LFC values using the unweighted pair group method with arithmetic mean method and a k-means k of seven. Seven clusters were selected through trialing different k values and reducing visible heatmap differences within clusters up to the point before clusters appeared similar. The resulting heatmaps were drawn with the R package ComplexHeatmap showing either LFC values for all RNAs in all of the RIP-seq experiments or the k-means center value for each cluster in each RIP-seq experiment. Transcript, CDS and UTR lengths were taken from our annotation set created from published datasets (18, 38). Translational efficiency was taken as untreated, Ribo-seq enrichment values (18). Published datasets were used for RNA half-life, SSU 5′UTR—start codon binding ratio and translational initiation (39, 45, 60). The proportion of cellular protein was calculated from the PaxDB whole organism integrated dataset (61). Theoretical classical translational efficiency was calculated using a tRNA adaptation index, which determines codon optimality based on tRNA gene copy numbers and codon usage in a subset of highly expressed genes (78, 79). The translational efficiency in an Asc1 mutant strain was taken from the asc1-M1X null mutant results (71). For each variable, each subset of data was compared to the all-data distribution with the Mann-Whitney *U* test and the *p*-values were adjusted with FDR correction. We considered statistically significant any adjust *p*-value < 0.05.

The R Bioconductor package clusterProfiler (103) was used to analyze the overrepresentation of annotation terms within the clusters. Gene ontology enrichment analysis was performed using GO slim mappings obtained from Saccharomyces Genome Database (https://downloads.yeastgenome.org/curation/literature/). Enrichment of cluster RNAs enriched in previous RIP-seq experiments was calculated using the “enricher” function for custom lists.

All sequencing data generated in this study have been submitted to ArrayExpress: (https://www.ebi.ac.uk/biostudies/arrayexpress/studies/E-MTAB-13033).

## Data availability

New RIP-seq data from this paper are available at Array express: E-MTAB-13033. Previous datasets from our lab used in this work are E-MTAB-2464, E-MTAB-5836 and E-MTAB-9095.

## Supporting information

This article contains supporting information (18, 36, 44).

## Conflict of interest

The authors declare that they have no conflicts of interest with the contents of this article.

## Supporting information

Supporting Figure S1 Polyribosome profiles of TAP-tagged strains used in this study where the positions of the ribosomal fractions (40S, 60S, 80S and polysomes), the direction of sedimentation and the absorbance (A_254_) axis are labelled (*A*). Western blot analysis of TAP-tagged proteins (*B*). Relative abundances of each protein are taken from a published dataset (36) (*C*). **Supporting Figure S2**Venn diagrams highlighting the degree of overlap between the mRNAs enriched with Pat1p and Lsm1p, and those found in P-bodies (*A*) and stress granules (*B*) from our published datasets (18). Venn diagram highlighting the overlap between the Ded1 enriched mRNAs and list of mRNAs with altered translation efficiencies in conditional *DED1* mutant strains (44). **Supporting Figure S3Gene Ontology (GO) analysis highlighting scale and significance of enrichments for the 7 clusters.** The analysis is depicted for the 3 different GO classifications: Biological process (*A*), Molecular function (*B*), and Cellular component (*C*). The numbers of mRNAs considered for each cluster and GO term are indicated in parentheses.

Supporting Table S1 A table showing raw sequencing read data for across new and past experiments.

Supporting Table S2 Tables showing the EdgeR fold change and False Discovery Rate derived from IP *versus* total mRNA sequencing data.

Supporting Table S3 Table depicting the mRNAs present in each of the 7 clusters detailed in Figure.
