# Supplementary figures and images for: Translation factor and RNA binding protein mRNA interactomes support broader RNA regulons for posttranscriptional control
Source: J Biol Chem. 2023 Aug 24;299(10):105195. doi: 10.1016/j.jbc.2023.105195 (PMC10562868; doi:10.1016/j.jbc.2023.105195)

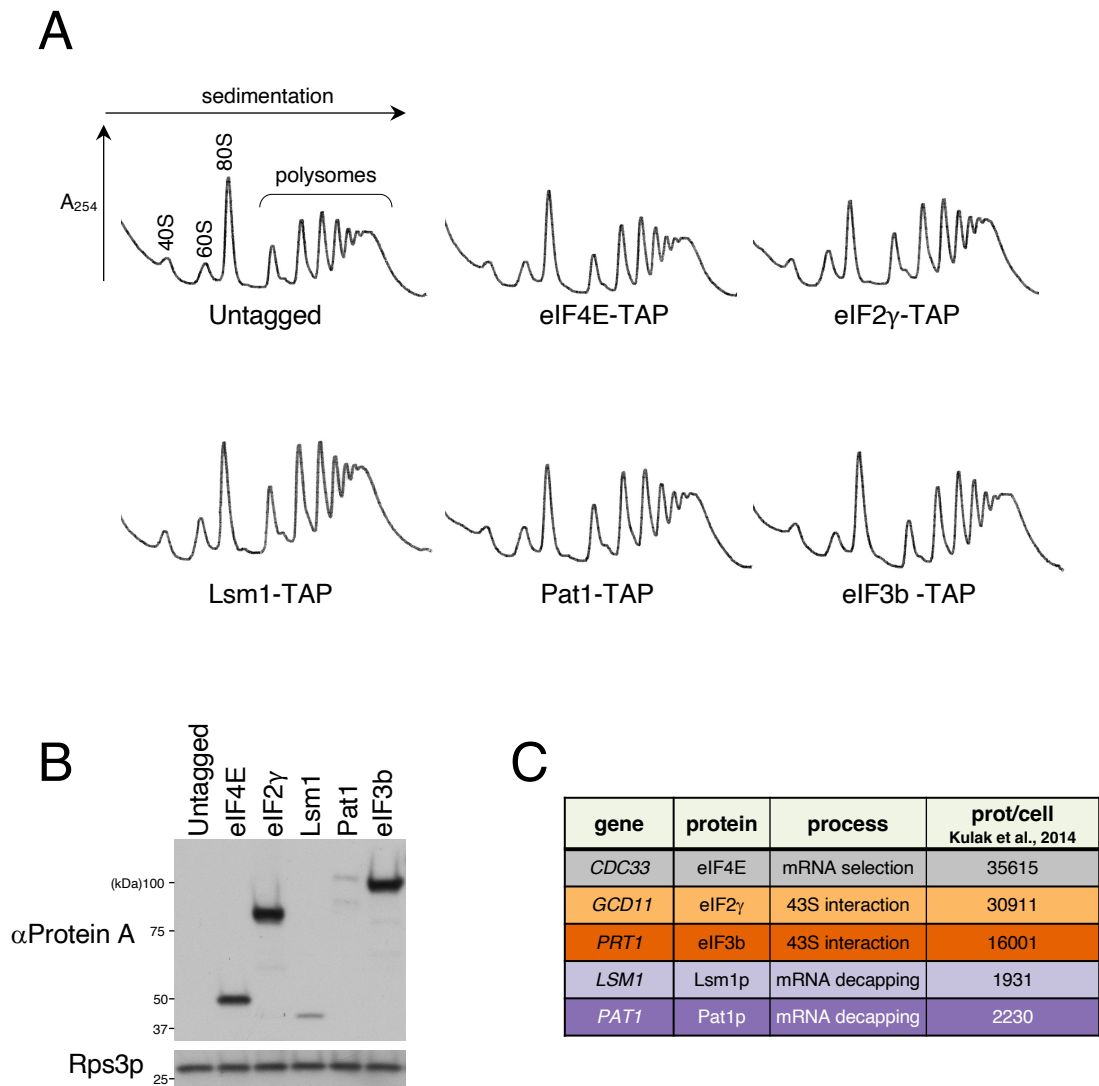

A

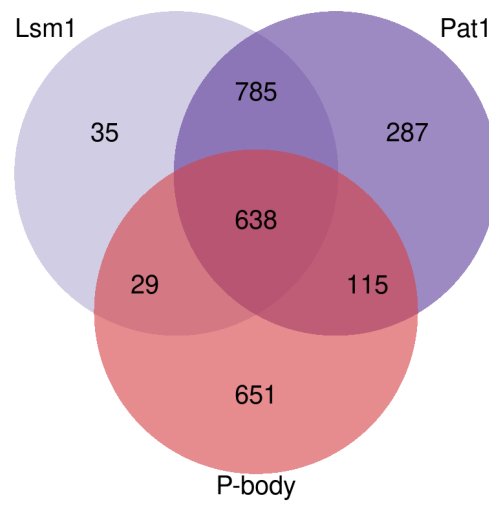

B

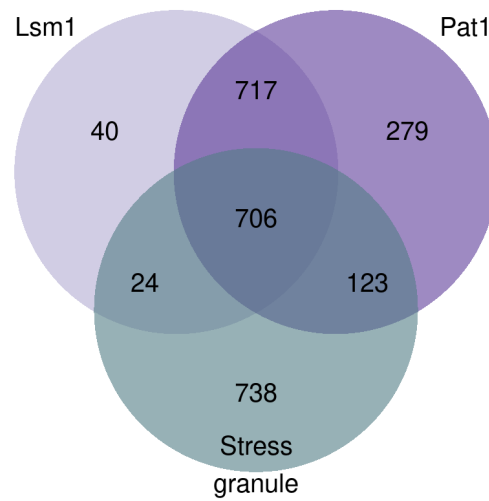

C

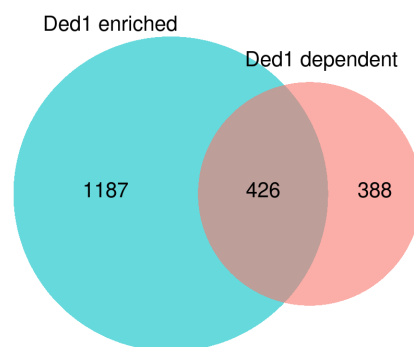

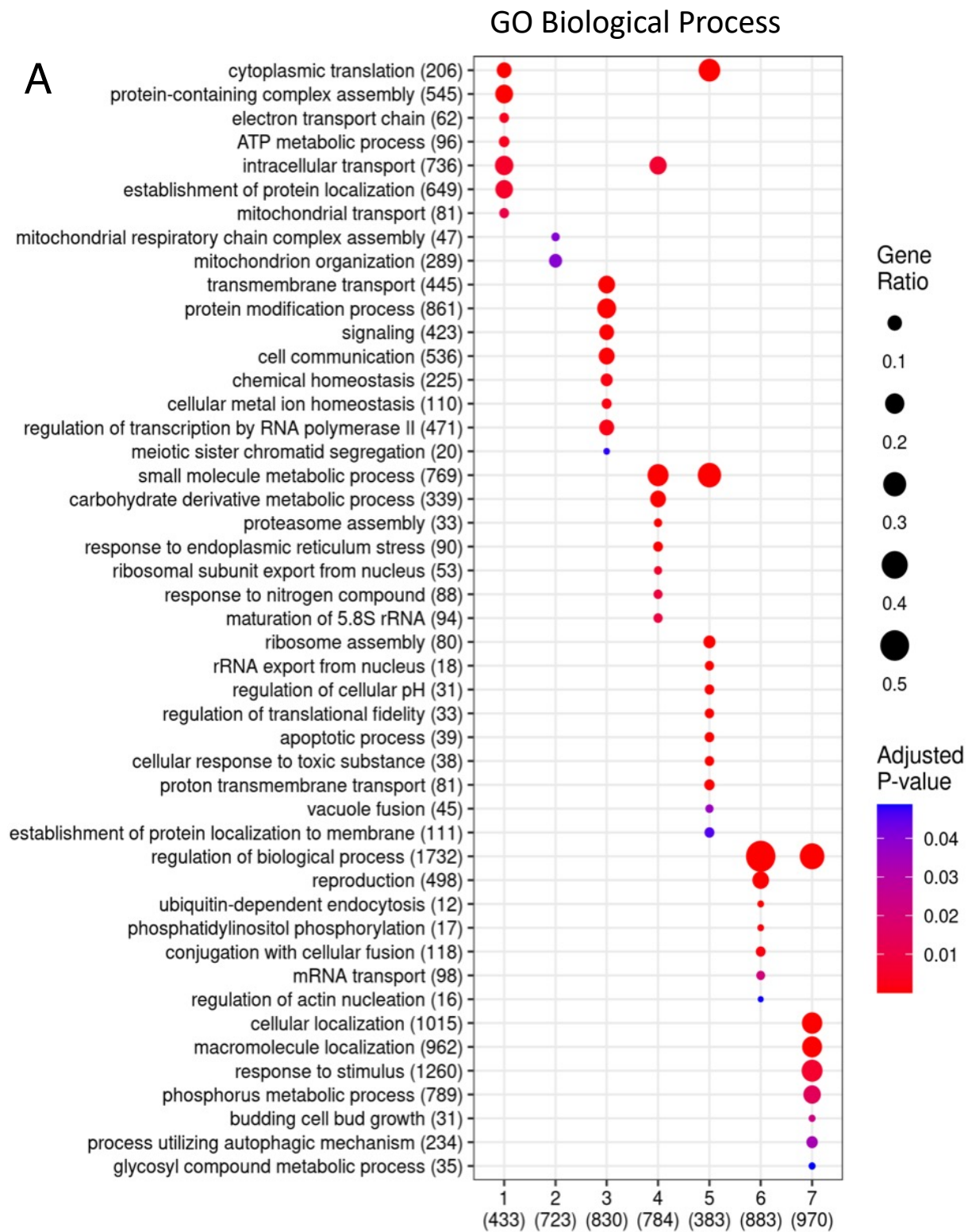

B

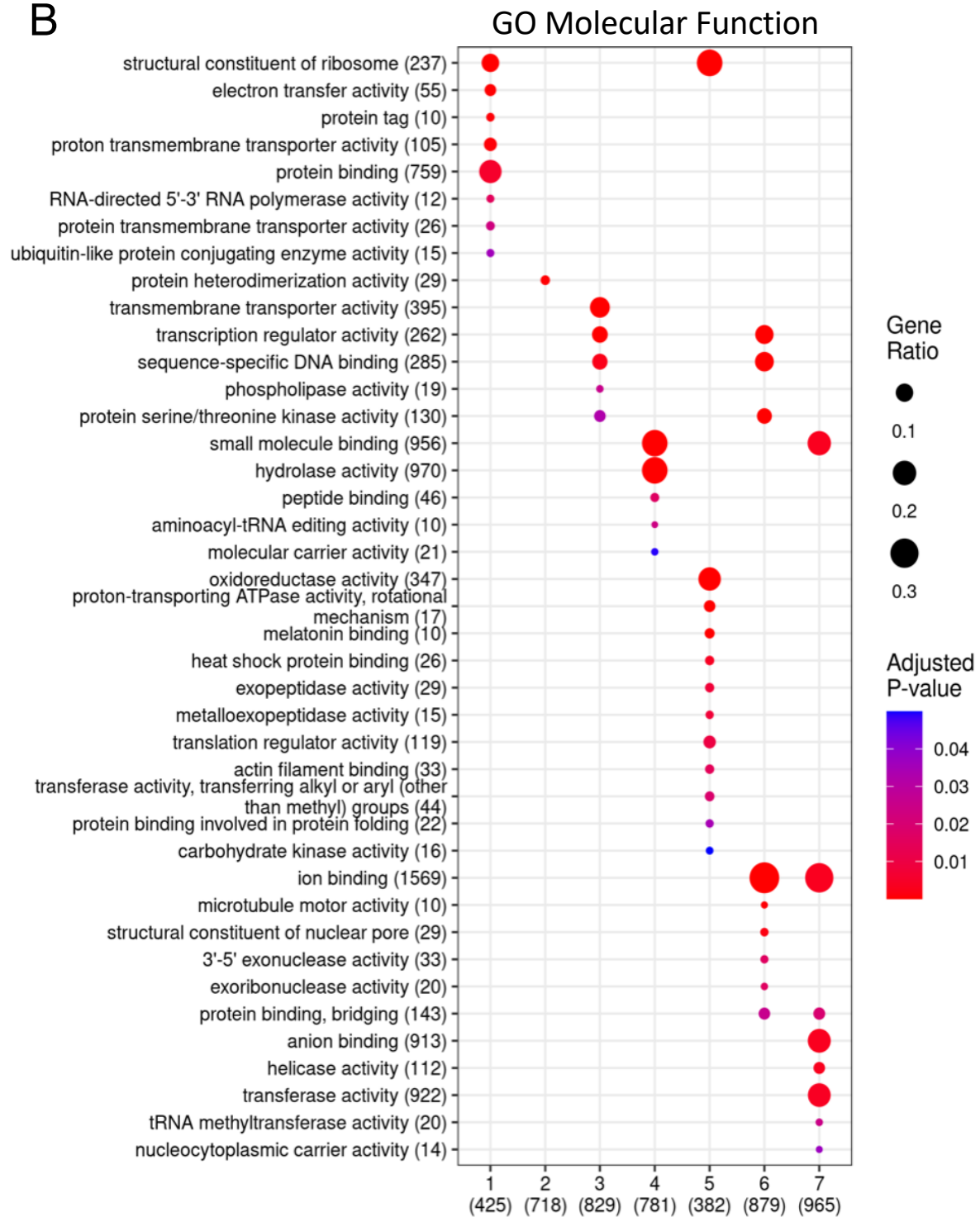

C

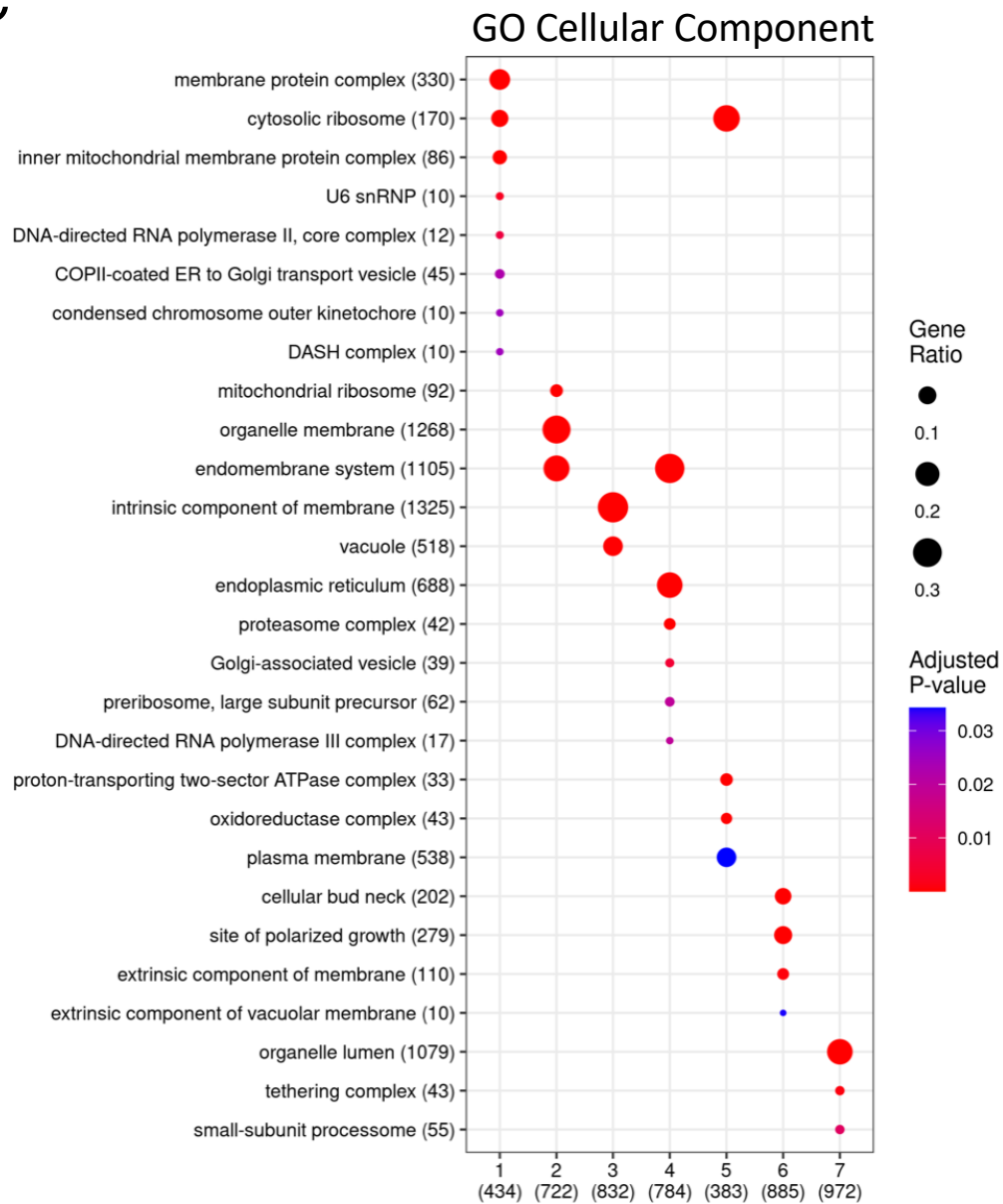

Supplement: Supporting Figure S1 — Polyribosome profiles of TAP-tagged strains used in this study where the positions of the ribosomal fractions (40S, 60S, 80S and polysomes), the direction of sedimentation and the absorbance (A254) axis are labelled (A). Western blot analysis of TAP-tagged proteins (B). Relative abundances of each protein are taken from a published dataset (36) (C). Supporting Figure S2Venn diagrams highlighting the degree of overlap between the mRNAs enriched with Pat1p and Lsm1p, and those found in P-bodies (A) and stress granules (B) from our published datasets (18). Venn diagram highlighting the overlap between the Ded1 enriched mRNAs and list of mRNAs with altered translation efficiencies in conditional DED1 mutant strains (44). Supporting Figure S3Gene Ontology (GO) analysis highlighting scale and significance of enrichments for the 7 clusters. The analysis is depicted for the 3 different GO classifications: Biological process (A), Molecular function (B), and Cellular component (C). The numbers of mRNAs considered for each cluster and GO term are indicated in parentheses. [file mmc1.pdf]
